# Patient Characteristics Associated With Disparities in Engagement With and Experience of COVID‐19 Remote Home Monitoring Services: A Mixed‐Methods Evaluation

**DOI:** 10.1111/hex.14145

**Published:** 2024-08-02

**Authors:** Nadia E. Crellin, Lauren Herlitz, Manbinder S. Sidhu, Jo Ellins, Theo Georghiou, Ian Litchfield, Efthalia Massou, Pei Li Ng, Chris Sherlaw‐Johnson, Sonila M. Tomini, Cecilia Vindrola‐Padros, Holly Walton, Naomi J. Fulop

**Affiliations:** ^1^ Research and Policy Nuffield Trust London UK; ^2^ NIHR Children and Families Policy Research Unit UCL Great Ormond Street Institute of Child Health London UK; ^3^ School of Social Policy, Health Services Management Centre, College of Social Sciences University of Birmingham Birmingham UK; ^4^ Institute of Applied Health Research, College of Medical and Dental Sciences University of Birmingham Birmingham UK; ^5^ Department of Public Health and Primary Care University of Cambridge Cambridge UK; ^6^ Department of Applied Health Research University College London Gower Street London UK; ^7^ Department of Targeted Intervention University College London London UK

**Keywords:** COVID‐19, disparities, health inequalities, home monitoring, patient engagement, remote monitoring

## Abstract

**Introduction:**

The adoption of remote healthcare methods has been accelerated by the COVID‐19 pandemic, but evidence suggests that some patients need additional support to engage remotely, potentially increasing health disparities if needs are not met. This study of COVID‐19 remote home monitoring services across England explores experiences of and engagement with the service across different patient groups.

**Methods:**

This was a mixed‐methods study with survey and interview data collected from 28 services across England between February and June 2021. Surveys were conducted with staff and patients and carers receiving the service. Interviews with staff service leads, patients and carers were conducted in 17 sites. Quantitative data were analysed using univariate and multivariate methods, and qualitative data were analysed using thematic analysis.

**Findings:**

Survey responses were received from 292 staff and 1069 patients and carers. Twenty‐three staff service leads, 59 patients and 3 carers were interviewed. Many service leads reported that they had considered inclusivity when adapting the service for their local population; strategies included widening the eligibility criteria, prioritising vulnerable groups and creating referral pathways. However, disparities were reported across patient groups in their experiences and engagement. Older patients reported the service to be less helpful (*p* = 0.004), were more likely to report a problem (*p* < 0.001) and had more difficulty in understanding information (*p* = 0.005). Health status (*p* = 0.004), ethnicity (*p* < 0.001), gender (*p* < 0.001) and employment (*p* = 0.007) were associated with differential engagement with monitoring, and minority ethnic groups reported more difficulty understanding service information (*p* = 0.001). Qualitative data found illness severity to be an important factor in the support required, and patients' living situation and social network affected whether they found the service reassuring.

**Conclusion:**

Addressing health disparities must be a key focus in the design and delivery of remote care. Services should be tailored to match the needs of their local population, encourage access through collaboration and referral pathways with other services and monitor their inclusiveness. Involving patients and staff in service design can illuminate the diversity of patients' needs and experiences of care.

**Patient or Public Contribution:**

The study team met with service user and public members of the BRACE PPI group and patient representatives from RSET in a series of workshops. Workshops informed study design, data collection tools, data interpretation and dissemination activities. Study documents (such as consent forms, topic guides, surveys and information sheets) were reviewed by PPI members; patient surveys and interview guides were piloted, and members also commented on the manuscript.

## 1 Introduction

In recent years, there has been a shift towards more remote models of healthcare. These models have been increasingly viewed as a potential solution to capacity issues confronting health systems. This shift was accelerated by the COVID‐19 pandemic, with the rapid adoption of digital technology changing healthcare delivery at an unprecedented pace [1, 2].

Remote home monitoring services have previously been implemented in the United Kingdom for chronic conditions [3, 4], increasingly adopted in the care of more acute illnesses [5, 6] (e.g., acute respiratory infections, chronic obstructive pulmonary disease and urinary tract infections) and hoped to be rolled out more widely for other conditions. Services for COVID‐19 patients were implemented in several countries [7] and were rapidly developed and scaled up in England between waves 1 and 2 of the pandemic (March 2020–January 2021) [8, 9, 10]. Patients using these services measured their oxygen saturation levels using a pulse oximeter to enable the remote assessment of their condition. Services aimed to ensure that patients were escalated earlier to avoid ventilation, intensive care admission and reduce unnecessary hospital attendance.

While there is limited research on patient experience and engagement with such services [7, 11, 12], remote models of care can place an additional burden on patients, and many patients require additional support to engage fully with such services [11]. Factors such as health, knowledge, support from family/friends and staff and informational and material resources can impact engagement [11]. However, little is known about whether and how remote care might affect existing health disparities [1, 2, 13, 14, 15], with benefits afforded by remote methods of healthcare delivery unlikely to be experienced by all patients.

Some patient groups are more likely to experience health inequities such as older adults, people with coexisting conditions, those with difficulties communicating, those isolated or experiencing language barriers and those with lower health literacy or understanding of how health systems work [14, 16, 17, 18, 19]. These patients may need additional support to access and/or engage appropriately with remote monitoring services [20]. Technology‐enabled remote methods (i.e., digital or automated monitoring) may exacerbate disparities in access and/or engagement, for example, due to digital exclusion (whether due to confidence using technology, digital access or literacy) [20, 21, 22]. Existing evidence [12] highlights the importance of mixed models of remote home monitoring (i.e., both a technology‐enabled and non‐technological option) to accommodate patient needs and encourage digital inclusivity. Understanding how vulnerable or at‐risk groups experience health services may help to reduce health disparities and shape healthcare delivery to become more accessible and effective [23].

Disparities can be introduced at any stage of the planning and delivery of health service interventions [24]. Evaluations of the effectiveness of strategies to reduce health disparities for remote monitoring services are scarce. Two studies on telehealth proposed the following: improving digital infrastructure for patients within/across organisations, collecting and monitoring data on technology access and literacy and codeveloping platforms [25, 26]. Strategies for reducing disparities in access and use of health services more generally include collecting and analysing data on access/use for relevant populations, redistributing/targeting resources in the population, outreach to and collaboration with communities where health disparities are known to exist (e.g., services in community spaces), using culturally and linguistically appropriate materials and training health professionals to share best practice [27, 28, 29, 30]. The effectiveness of strategies is largely unknown due to differences in reporting on health disparities and a lack of high‐quality studies [31].

Addressing disparities in healthcare access, quality and outcomes is high on the health policy agenda of many countries. In England, for example, the NHS Long Term Plan [32] has a significant focus on reducing health inequalities and addressing variation in care. To analyse whether disparities existed in how patients accessed, engaged with and experienced COVID‐19 remote home monitoring services and identify learning for developing and implementing inclusive remote models of care in the future, this evaluation addressed the following questions:

1. How were COVID‐19 remote home monitoring services adapted to facilitate inclusion and patient engagement for different groups?
2. Were there any disparities in relation to patients' engagement and experience of the services? If so, which patient characteristics were associated with these disparities?

## 2 Methods

### 2.1 Study Design

This multisite, mixed‐methods study was part of a larger evaluation of remote COVID‐19 home monitoring services [33]. The evaluation was carried out in England, UK, between February and June 2021 (see Supporting Information S1: Appendix 1 for detailed methods).

### 2.2 Conceptual Framework

We drew upon the National Institute on Aging (NIA) health disparities research framework to develop research questions and inform our analysis [34]. This framework was selected as it highlights a comprehensive range of factors that determine priority populations for health disparities research related to ageing. This was considered relevant to this programme as older adults were the primary target population of COVID‐19 remote monitoring services. We focussed our analysis on the following factors: gender, age, ethnicity, socioeconomic status, disability status and geographic factors—to examine disparities in relation to access, inclusivity, experience and engagement. We considered two additional factors: living situation and deprivation score (i.e., deciles of the Index of Multiple Deprivation).

### 2.3 Sampling and Data Collection

Twenty‐eight sites were selected using a range of criteria (i.e., setting, type of model, mode of monitoring, geographic location, timing of implementation). Following receipt of expression of interest, discussions were held with sites to collect information related to the sampling criteria (e.g., setting, type of model). We surveyed service leads and staff delivering the service and patients and carers receiving the service. A more in‐depth analysis of implementation and patient and staff experiences (through interviews with staff and patients) was completed at 17 of these sites (called ‘case study sites’). Case study sites were sampled using the same criteria as for the selection of study sites (outlined in Supporting Information S1: Appendix 1).

#### 2.3.1 Surveys

Online surveys administered to service leads and delivery staff focussed on staff perceptions of patient groups facing barriers to engagement and whether the service had been adapted locally to address any specific patient needs. Service leads coordinated the distribution of the patient survey (electronic or paper version) (see Supporting Information S1: Appendix 1 for inclusion criteria). The survey focussed on patients' experiences and engagement with the service including understanding the information provided, completion of tasks, whether they encountered problems and whether they would make any changes to the service (see Supporting Information S1: Appendix 2 for survey questions).

#### 2.3.2 Semi‐Structured Interviews

We carried out semi‐structured interviews (on the phone/MS teams) with 23 service delivery leads from 16 of the 17 case study sites. Interviews focussed on the design and implementation of services and staff experiences of implementation, including barriers and enablers.

Service leads from the 17 case study sites were asked to identify four to six patients to invite to participate in the study using a purposive sampling approach (see Supporting Information S1: Appendix 1 for inclusion criteria). To be inclusive and capture a wide range of views, service leads were asked to select patients with different characteristics (e.g., age, gender, ethnicity, deprivation score, employment status and comorbidities). Patient interviews focussed on how patients were referred to the service, how they felt about recording and monitoring their symptoms, how they communicated with the service and their experience of escalating care (see Supporting Information S1: Appendix 3 for topic guides). Patients were not asked directly about the accessibility or inclusiveness of the service or the impact of specific service adaptations.

Staff, patients and carers provided informed consent to participate.

### 2.4 Data Analysis

#### 2.4.1 Mixed‐Method Analysis

Quantitative survey data and qualitative interview data were collected concurrently from patients receiving the service and staff delivering the service. To triangulate findings, the interviews and surveys were analysed independently by four researchers (L.H., M.S., N.C., and H.W.) and then integrated. To illuminate the findings from the survey on different experiences of inclusiveness, a second round of analysis of patient interviews was conducted on the transcripts.

#### 2.4.2 Surveys

We analysed survey data using SPSS statistical software (version 25). Descriptive statistics were calculated to explore the number of services that made adaptations according to patient needs. Open‐text responses relating to service adaptations and patient groups facing barriers were analysed, triangulated with service lead interview data and coded into themes.

For patient survey data, we used descriptive statistics. Nonparametric univariate analyses were conducted to explore patient engagement with and experience of the service across patients grouped by different characteristics: age, gender, ethnicity, health status (i.e., whether experiencing a health problem/disability) education, employment status, English as the first language, living situation and deprivation score. We used Mann–Whitney *U* tests and Kruskal–Wallis *H* tests. Due to the large number of statistical hypothesis tests conducted and hence the possibility of false positive results at the traditional level of statistical significance (*p* < 0.05), a level of *p* < 0.01 was used.

We conducted logistic regression modelling to examine patient factors (including modality of the service, age, education, health status and ethnicity) associated with the likelihood of patients reporting a problem with the service. Patient open‐text survey responses providing feedback about the service were triangulated with patient interview data and coded into themes related to service design and engagement barriers.

#### 2.4.3 Service Lead and Patient Interviews

To understand the accessibility, suitability and effectiveness of services for different population groups, thematic analysis was carried out on service lead interview data from interview transcripts [35, 36]. Coding was conducted using NVivo 12 software and organised into themes related to service adaptations aimed at increasing access, inclusivity or engagement.

For patient interviews, data analysis was facilitated through the use of rapid assessment procedure (RAP) sheets [37]. RAP sheets contain a structured template to write key points from interviews in real time on a priori categories, based on questions included in the topic guide. To examine the inclusiveness of the services from the patients' perspectives, RAP sheets were analysed, and data were deductively coded using the framework outlined by the themes from the service lead interviews [37].

After we identified the differing experiences of the service from the staff and patient surveys, we returned to the transcripts of the patients' interviews to illuminate the findings. Specifically, we selected patients who were 75 years and over; living alone; of nonwhite British ethnicity (including those who spoke English as a second language) and/or had health problems that limited them (either a little or a lot). We examined their views related to areas identified as issues in the survey: reassurance from the service, information about the service, recording and communicating readings, support from family and friends and views of the service and support from staff.

## 3 Results

Twenty‐eight sites across England were included (see Table 1).

**Table 1:** Summary of site characteristics.

Site characteristic/domain	Number of sites (*n* = 28)	Number of case study sites (*n* = 17)
Type of model		
Covid oximetry at home^a^	13	9
Virtual ward^b^	4	1
Integrated Covid oximetry at home and virtual ward	11	7
Sector leading the service		
Primary care/community care	15	11
Secondary care	5	3
Both	6	3
Not specified	2	0
Mode of patient monitoring		
Analogue only^c^	7	3
Technology‐enabled and analogue^d^	21	14
Region		
London	5	3
South West	7	3
South East	5	4
North West	5	4
North East	2	1
East Midlands	2	1
East of England	1	1
Yorkshire and Humber	1	0
Month service started		
Before November 2020	11	7
In November 2020	12	9
After November 2020	5	1

^a^ Prehospital model.

^b^ Early discharge from the hospital model.

^c^ Paper and telephone.

^d^ A mixture of phone calls and tech‐enabled methods (i.e., app, web link).

### 3.1 Response Rates

We interviewed service delivery leads (*n* = 23), patients (*n* = 59), carers (*n* = 3) (see Supporting Information S1: Appendix 4 for demographics) and surveyed staff (70 managers/leads and 222 delivery staff, 39% response rate). We received 1069 surveys (18% response rate) from patients and carers across 25 sites (87.6%, 936 patients and 4.5%, 48 carers). See Supporting Information S1: Appendix 5.

### 3.2 Participant Characteristics

Table 2 presents patient survey respondent characteristics. We compared patient characteristics in our sample with those of all patients onboarded to COVID‐19 remote home monitoring services between October 2020 and April 2021 [38] to check the sample's representativeness with those engaging with the service. We found our sample was under‐representative of patients over 80 years (5% vs. 10%) and under 50 years of age (21% vs. 33%) and over‐representative of patients aged 50–80 years (74% vs. 57%). The survey sample comprised a higher proportion of patients from White ethnic groups compared to those onboarded to the service (91% vs. 76%) and a lower proportion of patients from minority ethnic groups. Finally, our sample was over‐representative of patients in the least deprived deciles of the Index of Multiple Deprivation (18% vs. 13%) and under‐representative of patients in the most deprived deciles (24% vs. 28%).

**Table 2:** Demographic characteristics for patient and carer survey respondents.

Demographic characteristic	Patient, *N* (%)	Carer, *N* (%)
Gender		
Female	531(58)	27 (60)
Male	385 (42)	18 (40)
Other/prefer not to say	4 (0.4)	0
Total	920 (100)	45 (100)
Age		
Under 50 years	195 (21.1)	13 (28.3)
50–64 years	428 (46.4)	24 (52.2)
65–79 years	256 (27.8)	4 (8.7)
≥ 80 years	43 (4.7)	5 (10.9)
Prefer not to say	1 (0.1)	0
Total	923 (100)	46 (100)
Living circumstances		
Living alone	132 (15.3)	
Household of 2	339 (39.3)	
Household of 3+	389 (45.1)	
Prefer not to say	3 (0.3)	
Total	863 (100)	
Ethnicity		
White^a^	836 (91.1)	38 (80.9)
Black^b^	16 (1.7)	0
Asian^c^	48 (5.2)	9 (19.1)
Mixed^d^	12 (1.3)	0
Other	2 (0.2)	0
Prefer not to say^e^	4 (0.4)	0
Total	918 (100)	47 (100)
Highest educational qualification		
No formal qualification	146 (16)	10 (21.7)
GCSE/CSE/O level or equivalent	273 (29.9)	16 (34.8)
A level/AS level or equivalent	106 (11.6)	8 (17.4)
Degree level or higher	212 (23.2)	7 (15.2)
Other	80 (8.8)	1 (2.2)
Prefer not to say/not sure	97 (10.6)	4 (8.7)
Total	914 (100)	46 (100)
Work situation^f^		
Working full time	355 (37.6)	17 (35.4)
Working part‐time	128 (13.5)	6 (12.5)
Self‐employed	41 (4.3)	0
Student in higher education	2 (0.2)	0
Unemployed	18 (1.9)	3 (6.3)
Homemaker/Full‐time carer	40 (4.2)	4 (8.4)
Retired	274 (29)	9 (18.8)
Furloughed	15 (1.6)	0
Not in work due to poor health or disability	65 (6.9)	5 (10.4)
Other/prefer not to say	31 (3.3)	1 (2.1)
Total	969 (100)	48 (100)
English as the first language		
Yes	852 (92.1)	35 (81.4)
No	66 (7.1)	8 (18.6)
Prefer not to say	7 (0.8)	0
Total	925 (100)	43 (100)
Day‐to‐day activities limited by a health problem or disability		
Limited a lot or a little	351 (38.1)	20 (43.4)
Not limited at all	482 (52.4)	17 (37)
Prefer not to say	4 (0.4)	1 (2.2)
Not sure/not applicable	83 (9)	8 (17.4)
Total	920 (100)	46 (100)
Deprivation score^g^		
1–2 (most deprived)	182 (23.7)	13 (35.1)
3–4	137 (17.9)	5 (13.5)
5–6	149 (19.4)	7 (18.9)
7–8	161 (21)	9 (24.3)
9–10 (least deprived)	138 (18)	3 (8.1)
Total	767 (100)	37 (100)

^a^ White British or any other White background.

^b^ Black, African, Caribbean, Black British or any other Black background.

^c^ Asian, Asian British or any other Asian background.

^d^ Mixed or multiple ethnic backgrounds.

^e^ Any other ethnic group.

^f^ Respondents able to select more than one option.

^g^ Deprivation by LSOA (Index of Multiple Deprivation decile).

### 3.3 How Were Services Adapted to Facilitate Inclusion and Patient Engagement For Different Groups?

Two‐thirds (16/24 sites) of the service leads surveyed reported that they had adapted their service to accommodate specific service user needs, including providing information in different languages, offering translation services, offering non‐digital monitoring options, face‐to‐face assessments and flexibility of monitoring methods. Staff service lead interviews and staff survey data indicated that there was considerable variation in the extent to which sites tailored services, for example, several services made substantial efforts to standardise the coverage of the service and invest resources into setting up pathways targeting hard‐to‐reach, vulnerable or at‐risk groups and to monitor uptake (see Tables 3 and 4 for full details of service adaptations to increase inclusivity and patient engagement).

**Table 3:** Service adaptations to increase inclusivity/reach.

Adaptations to service	
Broadening the service entry criteria	Using broader entry criteria than that specified in the national guidance to meet the needs of the local population, for example, minority ethnic groups, pregnant women and people with learning disabilities. Many services used an age criterion lower than 65 years (some adopted a 50+ cut‐off, while others used 18+). The broadening of criteria was reflected in national guidance, that is, allowing clinical judgement regarding assessment of entry criteria and under 65s permitted if clinically vulnerable.
Active case finding	Proactively identifying and contacting patients with a positive COVID test (rather than relying on referrals). Some services were able to do this from set‐up, other services introduced this later on (e.g., due to delays between Test and Trace linking positive cases to primary care services), while some services did not have the capacity to do this. Proactively targeting certain groups, for example, establishing a priority list for those patients considered to be hard to reach.
Design of referral pathways	Working with primary and/or secondary services to encourage appropriate referrals and improve the flow of referrals. Setting up additional referral pathways, for example, from emergency departments, 111, the ambulance service, out‐of‐hours services and care homes to increase referrals. Other, less frequent referral pathways were established including maternity wards, those for young carers, secure units and sheltered and supported accommodation. Services supporting, engaging and training health professionals based in primary care services to increase referrals.
Monitoring service uptake by different at‐risk groups	Regularly reviewing data on service uptake to check the representation of different at‐risk groups compared to local population data.

*Note:* The themes presented are derived from service lead interviews and staff survey data (i.e., open‐text responses).

**Table 4:** Service adaptations to increase patient engagement.

Adaptations to service	
Providing additional support for patients beyond monitoring protocol or national guidance	Connecting patients with other social support (and related services) in the local area. Providing more contact time to anxious, lonely or vulnerable patients or those living alone. Offering additional digital/technology‐related training or support to patients less confident or able to use technology. Locally providing additional support to help engagement with the service (e.g., to help with activities such as using the oximeter, recording and submitting readings). Providing wellbeing calls or welfare checks for vulnerable patients (i.e., telephone or face‐to‐face).
Collaborating with other teams or specialist services	Working with other clinical teams or specialist services, for example, learning disability teams or mental health services, respiratory or cardiac specialists and physiotherapists. Creating special/integrated pathways to better support patients with particular needs (including liaising with primary care services). Local variation in collaboration between the remote home monitoring service and other clinical services—patients in some areas had more seamless access to services, for example, to support existing health conditions or post‐COVID recovery.
Delivery of oximeters	Making arrangements for oximeters to be delivered to patients, where collection by a family member, friend or carer was not possible, for example, using volunteers or the fire service. Providing the option for oximeters to be collected by the patient (or friend/relative/carer) to ensure the fastest possible access to equipment. If patients are advised to use their own oximeter, safety netting information on how to use it is still required.
Adapting patient information	Providing information in different formats such as Braille or large print, easy‐read documents, and audio descriptions for patients. For example, easy‐read version of how to use the oximeter. Providing a link to an online video demonstrating how to use the oximeter. Providing information by post if no digital access. Liaising with family, friends or carers to support patients in understanding the information, or, if patients did not have support available to them, offering home visits. Recognising the importance of when patient information is given and to whom (i.e., patient and carers). To help patients and carers understand (as best as possible) what the service entails and what is expected of them.
Translation services	Amending patient information and supporting guidance for patients for whom English was not their first language, such as translating documents into other languages. Provision of information in accessible formats and translation of supporting information such as paper diaries into different languages. Providing translation services to translate information and support communication with patients. Liaising with family members, carers or friends to support non‐English speaking patients. Some digital platforms were able to support non‐English speaking patients.
Amending the monitoring processes/protocols	Changing the mode of monitoring, such as offering text options rather than telephone calls (e.g., if hearing impairment) or face‐to‐face appointments for those needing support with the equipment or where communication is difficult. Changing the timing of monitoring according to patient needs or preferences (e.g., around working hours or sleeping patterns). Changing the frequency of monitoring depending on preference or needs (e.g., less often if requested). Extending the monitoring period by allowing patients to remain in the service for longer (e.g., if still feeling unwell). For care homes, providing one phone call for all patients and calling at a routine time.
Mode of monitoring	Offering an analogue (rather than technology‐enabled) option for patients to record and submit their readings. All services offered an analogue mode of monitoring option regardless of whether they had a technology‐enabled option or not.
Providing additional equipment	Offering patients without access to technology, a tablet or similar to use to provide the readings throughout the duration of the service. Variation between services in whether they offered digital equipment and support.
Face‐to‐face assessments, home visits and/or video consultations	Offering patients face‐to‐face or home visits for those considered vulnerable or ‘at‐risk’ or those in which providing readings via phone or text is not possible (e.g., where communication is difficult via phone/text). National guidance specified that during triage face‐to‐face clinical assessment should take place if deemed necessary and discussions around support requirements should take place.
Liaising with family, friends or carer	Liaising with family members, friends or carers to provide readings for those who were too unwell to use the oximeter/provide readings, not confident with using the equipment or with a hearing or cognitive impairment.

*Note:* The themes presented are derived from service lead interviews and staff survey data (i.e., open‐text responses).

### 3.4 Were There Any Disparities in Relation to Engagement With And/Or Experience of The Service For Different Patient Groups?

In this section, we explore how different patient groups engaged with and experienced the service, focussing on accessibility of information, engagement with the service (particularly achievability of tasks) and problems reported (using patient survey data and patient feedback from interviews and open‐text survey responses, see Supporting Information S1: Appendix 6). Patient experience and engagement, more generally (i.e., not looking at differences across patient groups), are reported elsewhere [11].

#### 3.4.1 Accessing the Service

In interviews, patients reported being referred to the service by many different organisations and pathways. However, sites were not always consistent in the admission criteria applied; some patients reported inconsistencies in friends/family referred to the service, and several patients from sites that used active case finding reported that they did not know how they were referred. Collaboration between services was perceived to have facilitated more rapid access to care; several patients highlighted that they were supported quickly because of close liaison between the service and their GP, hospital or ambulance services. Once enroled on the service, there were some challenges relating to obtaining the appropriate equipment; some patients reported it was difficult to collect the oximeter when they were unwell and they relied on family or friends.

My husband had to go to the car park, because it was six thirty and they're closing the surgery. There's a nurse who handed it [the oximeter] over to my husband.

Site B, Patient 1

We had to go and get it [the oximeter] from the doctors. My sister went and got it from them.

Site G, Patient 6

#### 3.4.2 Accessibility of Information

Survey findings indicated variation across and between sites in the information available to patients and how it was provided. Older patients (*p* = 0.005) and those from minority ethnic groups (*p* = 0.001) reported more difficulty understanding information (due to limited sample size, it was not possible to examine differences between specific ethnic groups). Patient ratings of helpfulness of the information were also lower for older patients (see Table 5). In interviews, some patients with English as a second language reported difficulties with the information they were given, several indicated that they had relied on a family member to provide support in understanding the information and several reported that both verbal and written information would have helped given the difficulties they had in taking in the information, given that they were unwell.

If the hospital would just give us training, even on the last day, just to use the equipment and tell us how to do it exactly it would be nice… We did everything on the phone, so if they [the hospital staff] showed us then we would have been okay… if somebody can translate, like say how to do it, so if the person writes everything on their record in Gujarati or Hindu or whatever, so they know how to follow the link and how to use that.

Patient 2

**Table 5:** Summary of exploratory survey analysis examining differences in patient engagement with and experiences of services across groups.

Patient engagement	Summary of findings
Accessibility and helpfulness of information provided	Age Older patients reported less ease in understanding information about the service (*p* = 0.005); fewer patients aged 80 years and over reported understanding the information to be ‘very easy’ (40%) compared to all other age categories (60%–67%). Ratings of helpfulness of the information were lower for older patients (*p* = 0.005), with similar patterns of responses to understanding the information.
	Ethnicity Patients from minority ethnic groups reported less ease in understanding the information provided about the service (*p* = 0.001); 81% of patients from a minority ethnic group reported understanding the information to be ‘easy’ or ‘very easy’ compared to 96% from white ethnic groups.
Monitoring and escalation activities	Health status Patients with a health problem or disability reported more difficulty recording readings (*p* = 0.004). Fewer patients with a health problem or disability rated recording readings as ‘very easy’ (70%) compared to those without a health problem (79%), however when combined with ‘easy’ there was little difference between groups.
	Ethnicity Patients from minority ethnic groups reported more difficulty recording readings (*p* < 0.001). Fewer patients from minority ethnic groups reported recording readings (91%) to be ‘easy’ or ‘very easy’ compared to white ethnic groups (98%).
	Gender Male patients reported more difficulty providing readings to the service (*p* = 0.001). While similar numbers of males and females reported providing readings to the service as ‘easy’ or ‘very easy’ (when responses combined); fewer male patients reported providing readings (70%) to be ‘very easy’ compared to female patients (80%).
	Employment status Patients not in employment reported more difficulty with escalation activities (*p* = 0.007). Fewer patients who were not in employment reported seeking further help to be ‘easy’ or ‘very easy’ (84%) compared to those who were employed (89%).
Problems with the service	Age Higher age associated with an increased likelihood of reporting a problem.
	Level of education Higher level of educational attainment associated with an increased likelihood of reporting a problem.
Patient experience	Age Older patients reported the service to be less helpful (*p* = 0.004). Fewer patients aged 80 years and over reported the service to be helpful or very helpful (88%) compared to those in the under 50 years (92%) and 50–64 years age categories (92%).
	Employment status Patients in employment rated the service more positively (*p* = 0.001) and found it more helpful (*p* = 0.003) 96% in employment rated it as good or excellent and 93% as helpful or very helpful compared to 92% and 87%, respectively, among patients who were not employed.

Patient interviews identified several other factors affecting the accessibility of patient information. Patients with visual impairments reported that they would have welcomed information in a larger font, and several patients would have liked additional information about the service, their condition and recovery (e.g., information relating to the time of day to take readings, how to interpret oxygen readings, how to manage their recovery, symptoms to monitor, where to seek help).

#### 3.4.3 Engagement With the Service (Including Achievability of Tasks)

Most patient survey respondents reported monitoring and escalation activities as easy to engage with—the proportion reporting difficulties was low (see Table 5 and Supporting Information S1: Appendix 6). However, patient survey and interview data indicated that some patient groups might require additional support to engage with the service.

Patient survey respondents from minority ethnic groups reported more difficulty in recording readings (*p* = 0.001). Similar associations were found for providing readings to the service and seeking help but were marginally nonsignificant. Differences in ease of engagement across ethnic groups might be related to communication difficulties (see section 3.4.2), including English reading and writing ability which was needed to record readings using a tech‐enabled platform:

I'm not that educated and my English is good spoken wise, my reading is not that bad, but my writing is like a five year old child.

Site A, Patient 2

To tell the truth I was hopeless with computers so my, my daughter‐in‐law… did it every morning.

Site A, Patient 3

Also, staff and patient interviews indicated that translation services were often needed and not always available for patients for whom English was not their first language. Staff and patients reported that services often relied on liaising with friends or family to support communication, and this was not always possible.

Patient survey analysis found that male patients reported providing readings to the service as more difficult compared to females (*p* = 0.001). A higher proportion of males also reported the importance of friends and family in supporting their engagement with the service compared to females (25%; *n* = 97/385 vs. 19%; *n* = 102/531).

Older patients also reported that support from friends, family and healthcare professionals was more important for their engagement with the service. In the survey, 26% (*n* = 11/43) of patients over 80 years reported that they had support from family and friends to use equipment and 49% (*n* = 21/43) reported support from healthcare professionals helped them to engage with the service compared to 21% (*n* = 40/195) of patients under 50 years of age reporting support from family and friends and 43% (*n* = 83/195) reporting support from healthcare professionals. However, age differences in ease of engagement with monitoring activities were marginally nonsignificant. Patient interview data also indicated the value of support from friends and family members for older patients to engage with the service, with the need for support often related to health conditions, for example, arthritis making it difficult to record readings and hearing difficulties making it difficult to talk to the service via phone.

Patient survey respondents with a health problem or disability reported more difficulty recording readings (*p* = 0.004). Patient qualitative data also identified support from family and friends to take and provide readings, and liaise with the service was particularly crucial for patients who were more acutely unwell:

…[My daughter] did my food for me, she did all my medication, because I ended up having so much, we had one of those little boxes with the days and everything on it…. she'd speak to the service if I couldn't talk because I was coughing or breathless or whatever… Because I had [my daughter] with me as well… they knew that I had someone there who was reliable and responsible to ring if it got any worse.

Site C, Patient 4

Regarding patients' employment status, survey analysis of patient data found no statistically significant differences in engagement with monitoring activities. However, in interviews, several patients in employment reported they would prefer to reduce the number of readings per day or receive calls at a specific time each day, and survey analysis found patients who were not in employment reported seeking further help to be more difficult than those in employment (*p* = 0.007).

The survey analysis of patient data did not find any statistically significant differences in activities with deprivation scores or living situations. However, patients living with others reported greater reliance on support networks; 10% (*n* = 14/137) of patients living alone reported support from family or friends to engage with the service compared to 23% (*n* = 175/754) of those living with others.

#### 3.4.4 Problems With the Service

In the survey, 25% (*n* = 228/936) of patients reported at least one problem completing monitoring or escalation activities. Logistic regression analysis found that increasing age and a higher level of educational attainment were associated with an increased likelihood of reporting a problem (see Table 6).

**Table 6:** Multivariable logistic regression for patient‐reported problems to the service.

Variable	Participants	Participants reporting a problem (%)	Odds ratio	95% CI (lower–upper)	*p* value
Health status					0.727
Not limited at all	482	114 (23.7)	Ref.		
Limited a little or a lot	345	87 (25.2)	1.072	0.725–1.585	
Age					< 0.001
Younger than 50 years	195	40 (20.5)	Ref.		
50–64 years	428	95 (22.2)	2.295	1.314–4.009	0.003
65–79 years	256	71 (27.7)	2.910	1.606–5.273	< 0.001
80 years and over	43	15 (34.9)	7.639	2.869–20.339	< 0.001
Education					< 0.001
No formal qualifications	146	26 (17.8)	Ref.		
GCSE level or equivalent	267	46 (17.2)	1.338	0.728–2.460	0.348
AS level, A level, degree or equivalent	319	102 (32.0)	3.039	1.726–5.351	< 0.001
Ethnicity					0.081
White ethnic group	830	195 (23.5)	Ref.		
Minority ethnic group	74	23 (31.1)	1.794	0.931–3.457	
Mode of monitoring					0.123
Analogue	435	96 (22.1)	Ref.		
Tech‐enabled	501	132 (26.3)	1.363	0.920–2.021	

The odds of reporting at least one problem with the service were approximately 7.6 times higher for older adults (over 80 years) than younger adults (under 50 years). For patients aged 65–80 years, the odds were 2.9 times higher, and for patients aged 50–64 years, the odds were 2.3 times higher compared to patients under 50 years. The odds of patients who were educated to a higher level (i.e., AS, A level, degree level or equivalent) reporting at least one problem were three times higher than those of patients who had no formal qualification. The exploratory survey analysis did not identify any differences in ease of engagement with education, although differences in seeking help were marginally nonsignificant. Mode of monitoring, health status and ethnicity were not related to whether patients reported a problem.

#### 3.4.5 Experience of the Service

Overall, patients reported a positive experience of the service: 93% rated the service as good or excellent and 90% as helpful. Survey analysis indicated that patient‐reported experience of the service differed with age and employment status (see Table 5 for further detail). Older patients reported the service to be less helpful (*p* = 0.004) and reported a less positive experience of the service (although this was not statistically significant). However, patient interview data indicated that many found the service to be reassuring. Patients in employment rated the service more positively (*p* = 0.001) and found it more helpful (*p* = 0.003), which might be explained by differences in ease of seeking help (reported earlier) or might be related to other factors, such as health status, education or age, with older patients more likely to be retired. Living situation was not identified in the survey analysis to be a significant factor in patient experience. However, patient interview data indicated living situation and strength of social network as important; reassurance provided by the service was reported by patients to be particularly important for those socially isolated or living alone:

Just comforting [speaking with the nurses] because obviously I'm living alone, so it was just that really, I knew somebody was watching out for me.

Site E, Patient 6

## 4 Discussion

### 4.1 Key Findings

Many service leads reported adapting their service to be more inclusive to the needs of their local population to expand its reach and inclusivity and facilitate engagement. Strategies identified included broadening service inclusion criteria for at‐risk groups, setting up referral pathways across services, providing information in different languages or formats, offering translation services, offering analogue and tech‐enabled options, face‐to‐face assessments, providing additional training or support, flexible monitoring processes and liaising with family or friends. There was considerable variation in the adaptations employed by services to meet the needs of specific patient groups. However, the involvement of service users, carers and/or community groups in the design of service adaptations was rarely discussed.

Despite this, our exploratory analysis identified differences across patient groups in their experience and engagement with the service. Older patients (over 80 years) reported the service to be less helpful and were more likely to report a problem with the service. They reported requiring more support from friends, family and health professionals to engage with the service and more difficulty in understanding the service information. Health status, ethnicity, gender and employment were associated with differential engagement with monitoring and escalation activities. Patients with a health problem or disability reported more difficulty completing monitoring activities, and qualitative data indicated that the severity of illness was an important factor in whether patients were well enough to engage/how much support was required. Patients from minority ethnic groups reported more difficulties engaging and understanding service information. Translation services were often needed and, however, were not always available, leading services to rely on friends or family for support. Findings extend our published work exploring patient experiences of the service by focussing on the diversity of patient experience and unmet support needs [11, 12].

### 4.2 How Findings Relate To Previous Research

Previous evaluations have investigated models of remote home monitoring and implementation of COVID‐19 services; however, to our knowledge, this is the first study to explore service adaptations adopted to address disparities in patients' access, engagement and experience. Our findings support previous international research on strategies that can be used to reduce disparities in access to and use of health services more broadly, such as targeting resources to at‐risk groups by expanding admission criteria and referral routes and ensuring digital inclusion by offering analogue monitoring options [25, 27, 30]. Our findings extend research by outlining additional strategies to address disparities by providing additional support beyond monitoring, for example, face‐to‐face visits, providing more contact time to anxious or vulnerable patients, offering additional digital‐related training or support to those less able or confident using technology, connecting patients with other local support services, amending protocols to patient need and liaising with family or carers. Other strategies reported within the wider international literature as used to address potential disparities include routinely collecting and analysing data on populations, technology access and digital literacy to check the representativeness of uptake and monitor impact on disparities, co‐developing platforms with patients and staff and working with communities where disparities are known to exist [25, 26, 27, 28, 29, 30, 39, 40].

Recent work [41] conducted in primary care in the United Kingdom outlined five principles for interventions to reduce inequalities as follows: (i) connected services, (ii) an intersectional perspective (accounting for differences within patient groups), (iii) flexibility in responding to patient needs and preferences, (iv) inclusive and (v) community centred in engaging communities with service design and delivery. Many of the strategies adopted by remote monitoring services correspond to these principles (e.g., collaboration across services, tailoring to patient needs); however, few sites reported the involvement of service users and/or community groups in the design and delivery of services.

This manuscript extends our work examining patient experiences of remote monitoring models of care [11, 12] by exploring whether engagement and experiences differ across patient groups. There is no existing evidence focusing on the impact of such services on different patient groups and health disparities. Our findings, however, support research (conducted within the United Kingdom and United States) that has demonstrated similar trends in patients' experiences of accessing and using health services more broadly ‐ relating to ethnicity and age, and considering differences in expectations, health literacy, digital skills, staff‐patient communication and understanding how systems work [19, 42, 43]. For example, reported differences with age might be explained by experiences of engaging digitally—a recent international review found older adults to report more challenges engaging with digital health interventions [44].

Our finding that a higher level of educational attainment was related to an increased likelihood of reporting a problem contradicts international literature evidencing links between higher education and improved health literacy and healthcare experiences [45] but is perhaps due to other factors such as differing expectations of the service, confidence in advocating for own needs or knowledge around best practice or availability of services. Our finding that there were no differences in engagement or experience of the service according to the level of deprivation contrasts previous (UK and international) findings that those from more deprived groups tend to demonstrate a lower level of engagement and participate less actively with their healthcare more generally [46, 47]. However, it is important to note that our sample was under‐representative of patients in the most deprived deciles.

Some patient groups required additional support from friends, family or health professionals to engage with the service—particularly those who were unwell, living alone and older patients. The importance of family and social support when implementing remote monitoring models of care has previously been evidenced [5]. The accessibility of information provided about the service might, in part, account for some differences in patient experiences—older patients and those from minority ethnic groups reported less ease in understanding information. Previous research conducted in the UK shows that communication, particularly receipt of clear and accessible information, is key in maximising patients' experiences of care—poor communication and language barriers have been found to impact the relationship between patients and health service providers [48]. Other potential explanations for observed differences might be related to health literacy and expectations about receiving good healthcare. For example, poorer health literacy has been previously evidenced (within research conducted in the UK) among older adults and those with lower educational attainment and lower socioeconomic status [49], and expectations of care have been found to differ across patient groups [50].

### 4.3 Implications

As the transformation of health services continues towards more remote models of care, addressing health disparities must be a key focus in their planning, design and delivery. Our evaluation generates a range of recommendations for the design and delivery of inclusive remote monitoring services that are applicable across a range of health conditions. More specifically, learnings can support the implementation of the NHS virtual wards programme [51] (such as for acute respiratory infection, frailty) within the United Kingdom, as well as broader remote monitoring programmes delivered internationally for a range of conditions. For example, helping to consider how patient information should be provided, how models are delivered to address the needs of different populations (i.e., use of technology, flexibility, ways of communicating, ease of engagement) and how the implementation of such models is monitored relating to potential inequalities (i.e., the need to collect and monitor appropriate data). Differences in engagement with and experiences of remote care across patient groups demonstrate that services must consider the needs of their local population and adapt accordingly, work with existing local systems (e.g., community groups and local authorities) to engage hard‐to‐reach groups, encourage access through collaboration and linking with other services and monitor the inclusiveness of the service (particularly the impact on population groups at risk of health inequities).

As patients' ability to engage with remote care varies, patients' needs and circumstances should be assessed in a standardised way upon referral and services tailored to provide appropriate levels of support for patients and carers. Examples of tailoring include patient information in a range of formats to increase accessibility [23, 52], additional information, education or support to use equipment or in navigating services, flexible and personalised monitoring processes and a non‐digital option for patients without relevant digital skills or infrastructure. Appropriate tailoring of health interventions can promote health equity and is key for responding to individual needs [53], and additional support is important for patient groups experiencing difficulties accessing or engaging with systems [20]. Where relevant, services should encourage the involvement of family, friends or carers but should provide adequate tools and support to reduce the burden placed upon them [54]. Appropriate training and support should be provided to healthcare professionals to develop effective communication strategies to facilitate tailored, culturally sensitive care [48].

When implementing remote models of care (whether NHS virtual ward or remote monitoring more broadly), adequate resources and infrastructure should be provided to allow patients to be involved in the design and implementation. Codesign can help to ensure services are better suited to patient needs and facilitate engagement [55]. Input should include representation across patient groups [48, 56]. Involvement and representation across services can help to facilitate collaboration and linking between services to generate the flexibility to respond to patient needs [53, 55].

### 4.4 Strengths/Limitations

Our research has several strengths. The mixed‐methods design allowed for the triangulation of data across data sources. The evaluation was conducted across several sites, and the large number of staff and patients sampled increased the generalisability of findings. The evaluation team was multidisciplinary, which facilitated triangulation and interpretation.

Several patient groups were under‐represented in the survey when compared to national onboarding data, and the response rate for the patient survey was relatively low (18%); so, some impact of selection bias cannot be ruled out. Findings might not be representative of all patient groups and experiences, such as those not referred, declined or disengaged from the service. The analysis of experience and engagement across patient groups was exploratory and could have been subject to false positives due to the number of comparisons made, although a more stringent p‐value was used (*p* < 0.01) in reporting significant results. When making comparisons between patient groups, the sample size of several groups was relatively small (e.g., patients aged over 80 years). Given that the study was part of a larger evaluation of COVID‐19 remote home monitoring services, patient interviews focussed on experiences of the service more broadly and not necessarily disparities; so, much of our analysis draws on staff perspectives or patient survey data.

### 4.5 Future Research

There is little published literature on the implementation of remote monitoring and health disparities. Our exploratory analysis helps to understand some of the disparities that might exist in patients engaging with remote monitoring services; however, further evaluation is needed to better understand how and why experience of and engagement with services differ across patient groups. There are other populations at risk of health disparities and patient characteristics that were beyond the scope of this evaluation that should be considered in any future evaluations—for example, the homeless community, people with disabilities, people with a mental health diagnosis and people with a visual or hearing impairment. Urban versus rural living should also be explored in considering engagement with such services. The scope of our evaluation did not allow us to examine the impact of specific strategies made by local services to increase accessibility or engagement. Therefore, we are unable to make any conclusions about their effectiveness nor are we able to determine the workforce or resource implications of specific strategies; this is an area for future research.

## 5 Conclusion

When health services undergo rapid transformation, evaluations of the effectiveness of such services must include an impact assessment to ensure the service is accessible and inclusive to all patients and to monitor the impact on health disparities. Our findings demonstrate that not all patients experience remote monitoring services equally, and addressing health disparities must therefore be a key focus in the planning, design and delivery of remote monitoring models to ensure they are of value to all population groups. Services should be tailored to match the needs of the population they serve, and staff and patients from groups typically experiencing disparities in their healthcare must play an active role in service design to ensure their needs, experiences and expectations are accounted for. More work is urgently needed to understand access, engagement with and experiences of remote monitoring services for different population groups.

## Author Contributions

**Nadia E. Crellin:** formal analysis, writing−original draft, methodology, writing−review and editing, project administration, data curation. **Lauren Herlitz:** formal analysis, writing−original draft, methodology, project administration, writing−review and editing, data curation. **Manbinder S. Sidhu:** writing−original draft, formal analysis, methodology, writing−review and editing, project administration, data curation. **Jo Ellins:** writing−review and editing, methodology, supervision, writing−original draft, funding acquisition. **Theo Georghiou:** writing−review and editing, writing−original draft, methodology, supervision. **Ian Litchfield:** writing−original draft, writing−review and editing, project administration. **Efthalia Massou:** writing−original draft, writing−review and editing, methodology. **Pei Li Ng:** writing−original draft, writing−review and editing, project administration. **Chris Sherlaw‐Johnson:** writing−original draft, writing−review and editing, methodology, supervision. **Sonila M. Tomini:** writing−original draft; writing−review and editing, methodology. **Cecilia Vindrola‐Padros:** writing−original draft, writing−review and editing, methodology, project administration. **Holly Walton:** data curation, formal analysis, writing−original draft, writing−review and editing, project administration, methodology. **Naomi J. Fulop:** funding acquisition, supervision, methodology, conceptualization, writing−original draft, writing−review and editing.

## Ethical Statement

The staff aspects of the evaluation were categorised as a service evaluation by the HRA decision tool and UCL/UCLH Joint Research Office and received ethical approval from the University of Birmingham Humanities and Social Sciences ethics committee (ERN_13‐1085AP39). The patient aspects were reviewed and given favourable opinions by the London‐Bloomsbury Research ethics committee (REC reference [21]:/HRA/0155).

## Conflicts of Interest

The authors declare no conflicts of interest.

## Supporting information

Supporting information.

## Data Availability

Due to the consent process for data collection within this evaluation, there are no data that can be shared.
